# Comparative Morphology and Ultrastructure of Antennal Sensilla in *Dendrolimus superans* (Lepidoptera: Lasiocampidae) and *Lymantria dispar* (Lepidoptera: Lymantriidae)

**DOI:** 10.3390/insects15090655

**Published:** 2024-08-30

**Authors:** Qi Wang, Longzheng Wang, Qing Wang, Shanchun Yan

**Affiliations:** 1 Key Laboratory of Sustainable Forest Ecosystem Management, Ministry of Education, Northeast Forestry University, Harbin 150040, China; qiqi8325@163.com (Q.W.); wanglzchina@163.com (L.W.); 15776488980@163.com (Q.W.); 2 Forest Protection Research Institute of Heilongjiang Province, Heilongjiang Academy of Forestry, Harbin 150040, China

**Keywords:** *Dendrolimus superans*, *Lymantria dispar*, antennal sensilla, ultrastructure, scanning electron microscopy

## Abstract

**Simple Summary:**

Insects’ antennae are considered to be the principal organs of insects’ olfactory sensory systems, with their surfaces harboring a vast array of specialized and differentiated receptors. In this study, we compared the morphology and ultrastructure of the antennal sensilla of *Dendrolimus superans* and *Lymantria dispar* using scanning electron microscopy (SEM). Furthermore, we conducted a comparative analysis to assess the differences in sensillum characteristics, quantity, and distribution between *Dendrolimus superans* and *Lymantria dispar* male and female individuals.

**Abstract:**

*Dendrolimus superans* (Lepidoptera: Lasiocampidae) and *Lymantria dispar* (Lepidoptera: Lymantriidae) are two important forest defoliators in northeast China, with the former being a specialist on *Larix* spp. and the latter being a generalist feeding on >500 species of plants. The morphology and ultrastructure of antennal sensilla of both male and female *D. superans* and *L. dispar* were examined using scanning electron microscopy (SEM). In both sexes of *D. superans*, the following five types of antennal sensilla were found: sensilla trichoidea, s. chaetica, s. coeloconica, s. gemmiformia, and s. basiconica. In males of *L. dispar*, six types of antennal sensilla: sensilla trichoidea, s. chaetica, s. coeloconica, s. basiconica, s. styloconica, and s. auricillica, were identified. In addition to the six types found in males, a seventh type of sensilla, s. squamiform, was only detected on *L. dispar* female antennae. For s. chaetica of *D. superans*, a unique ultrastructure of sub-branches that have one branch, two branches, and three branches was observed on their tips, which has not yet been reported on other insects. s. styloconica, s. auricillica, and s. squamiform, not found in the specialist *D. superans*, may be related to the euryphagy of *L. dispar*. Potential functionalities of these sensilla were discussed with reference to moth feeding habits, and their morphology, distribution, and ultrastructures on both species.

## 1. Introduction

Antennae are the most important sensory appendages of insects and carry different types of sensilla [1,2] for olfaction, gustation, mechanoreception, and thermo- and hygroreception [3,4,5]. Antennal olfactory sensilla are crucial for mate finding, host plant recognition and selection, and self-defense in insects [6,7]. Currently, their structures and functions have been the subject of numerous investigations using scanning electron microscopy (SEM) [2,8,9,10,11,12,13,14].

Different insect species possess diverse sensilla types in different relative abundances. Lepidoptera, the second largest order of insects [15], may have tens of thousands of sensilla, which are classified into several subsets of morphological types [2,16,17]. The olfactory sensilla of Lepidoptera insects are mainly distributed on their antennae, and according to their external morphology, they mainly include the following: sensilla trichoidea, sensilla basiconica, sensilla coeloconica, sensilla chaetica, sensilla auricillica, sensilla placodea, and sensilla styloconica [2,18]. Each type of sensilla has different specific functions; therefore, to understand the olfactory behaviors and recognition mechanisms of insects, it is necessary to identify the types, distributions, and functions of antennal sensilla and to study the morphology and structure of olfactory sensilla [19].

*Dendrolimus superans* (Lepidoptera: Lasiocampidae) and *Lymantria dispar* (Lepidoptera: Lymantriidae) are two serious forest pest insects with a great economical and ecological significance in Northern China [20,21]. The major effective method to control both pests still relies on chemical pesticides, which not only results in environmental pollution, but also has bad effects on human health [22,23]. So, efficient and environmentally sound control measures for devastating both of these pests are urgently needed. However, little is known about the morphology and distribution of antennal sensilla in *D. superans* and *L. dispar*, or their role in basic biological and ecological functions. This has greatly restricted the development and application of insect olfactory control methods.

Moreover, *D. superans* is a typical oligophagous pest insect, mainly feeding on needles of *Larix* spp., while *L. dispar* is an euryphagous pest, foraging on more than 500 species of plants or trees [24]. There are significant differences in the host plant range between these. Antennae are used for the recognition of host plants. Therefore, in the study of such pest species, it is very important to know the sensory systems of their antennae [25]. The differences in host diversity between them may be related to the number, structure, and function of their sensilla, but there are no reports on antennal sensilla of these two species to explain this issue.

In this study, we compared the morphology of the antennal sensilla of these two economically important moth species, using scanning electron microscopy (SEM). Such knowledge would provide some basic information for better understanding the functions of antennal sensilla and receptor neurons and establish a basis for further research into insect–insect or plant–insect pheromone interactions. These results may also provide theoretical guidance for the development of environmentally sound insect pest management technologies.

## 2. Materials and Methods

### 2.1. Insects

The pupae of *D. superans* were collected in March 2023 from Keshiketengqi, Inner Mongolia, P. R. China, while *L. dispar* eggs were obtained from the Forestry Centre of Northeast Forestry University in Harbin, Heilongjiang, P. R. China; and both were reared in the laboratory at 25 ± 2 °C, 75 ± 5% RH and in a 14L:10D h photoperiod until the adults emerged. The adult moths were separated by sex right after their emergence.

### 2.2. Scanning Electron Microscopy

Before the SEM (scanning electron microscopy), FEI, Hillsboro, OR, America, each freshly cut antenna was cleaned in an ultrasonic bath for 5 s with alcohol and then dehydrated by placing it into a beaker through a graded alcohol series of 50%, 70%, 80%, 90%, 95%, and absolute ethanol twice, each for 15 min before air drying. Thereafter, samples were mounted on aluminum stubs using double-sided adhesive tape and sputter coated with gold. Examinations were conducted with a FEI-QUANTA-200 SEM (FEI, Hillsboro, OR, USA) at 15 kV. The antennae of both sexes were examined in the same fashion. Micrographical images of both the antennae and sensilla were taken, and the dimensions of the sensilla were measured. The abundance and distribution of each antennal sensilla type were recorded. The morphological terms used here followed those of Schneider [26], Zacharuk [4], and Keil [27]. The antennal morphology and sensilla number for each segment, and the length, diameter, and density of those sensilla, were directly measured from the printed SEM images.

## 3. Results

### 3.1. Antennal Morphology and Sensilla Types of D. superans

**Figure 1:**
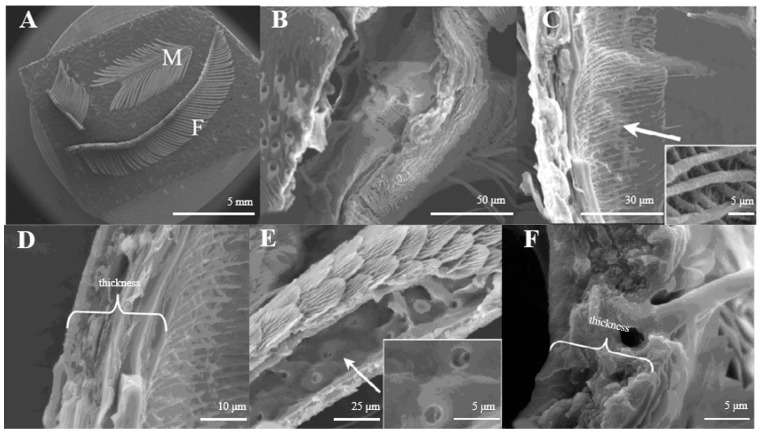
(**A**) Morphology of *D. superans* antennae; M: male; F: female; (**B**) the principal antennal axis (×800); (**C**) the inner structure of principal antennal axis (×1000, ×10,000); (**D**) the wall of antennal principal axis (×2500); (**E**) the inner structure of antennal branches (×1500, ×5000); (**F**) the wall of antennal branch (×5000).

The antennae of male moths are plumose and those of females are pectinate. There are no obvious confines between the scape and pedicel; the flagellum is composed of several sub-segments, and among them, the male flagellum is composed of 48–56 sub-segments and the female is 69–76 sub-segments (Figure 1A). In both sexes, all segments are covered with sensilla, on which pores or squamae were detected.

The principal axis of the antenna looks like a pipeline (Figure 1B) inner structure with a reticular (Figure 1C). On average, the axis wall is about 10.5–10.8 μm in thickness (Figure 1D). The antennal branches are tubular (Figure 1E), too. The difference between the antennal axis and antennal branches is that the inner structure of the axis is reticular, while those of the antennal branches are smooth with small pores (Figure 1C,E). The wall of antennal branches is 8.2–8.3 μm in thickness (Figure 1F).

In both sexes, the antenna has an even diameter throughout its length. The following five types of antennal sensilla are present in both sexes: sensilla trichoidea, s. chaetica, s. coeloconica, s. gemmiformia, and s. basiconica.

#### 3.1.1. Morphology 

##### Sensilla Trichoidea (ST)

**Figure 2:**
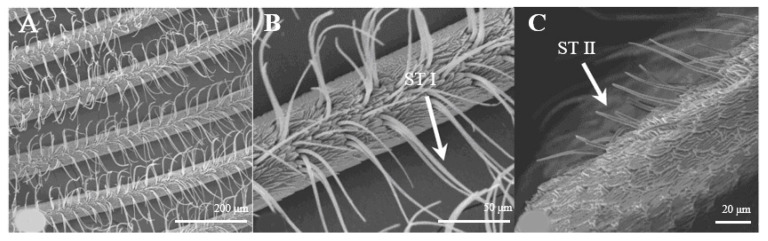
Morphology of sensilla trichoidea in *D. superans*. (**A**) s. trichoidea on antenna (×200); (**B**) s. trichoidea I on antenna (×800); (**C**) s. trichoidea II on antenna (×1000).

Sensilla trichoidea are the most abundant types on male antennae, especially on the windward side of antennal branches. s. trichoidea are hairs and have a slight curvature. They are divided into two sub-types, s. trichoidea I and s. trichoidea II, according to their length and shape (Figure 2A). s. trichoidea I (Figure 2B) are 62–64 μm in length, with their basal diameters being 1.3–1.4 μm, standing vertically on the antennal branches in clusters of five–six sensilla (Table 1). The s. trichoidea II (Figure 2C) are located on the base of female antennal branches or among s. trichoidea I, and they are 23–25 μm in length and 2.2–2.3 μm in basal diameter. On the female antennae, ten branches with a length of about 1.5 mm are randomly selected, and the average number of s. trichoidea I and s. trichoidea II on each branch is 536 ± 47 and 231 ± 23, respectively. The numbers of s. trichoidea II are much less than those of s. trichoidea I.

##### Sensilla Chaetica (SCh)

**Figure 3:**
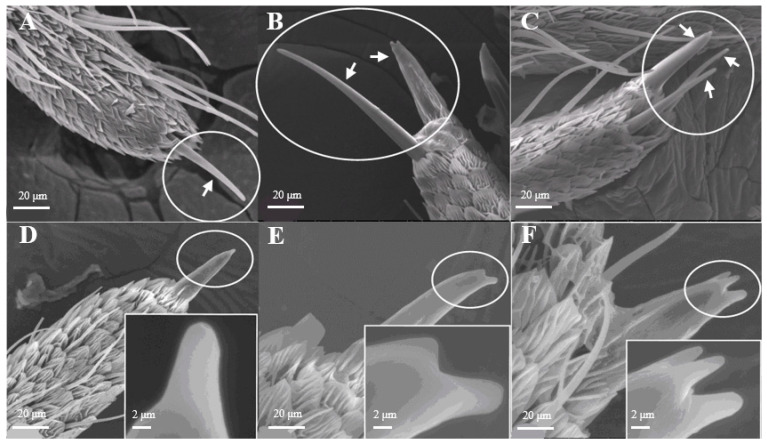
Morphology of sensilla chaetica in *D. superans*. (**A**–**C**) s. chaetica on the tip of antennal branch (×1000); (**D**–**F**) the spike (spine) of s. chaetica (×1000, ×10,000).

Most of the Sensilla chaetica (Figure 3) have three sub-types, such as one branch (Figure 3A), two branches (Figure 3B), and three branches (Figure 3C). Its unique structure has not yet been reported on other insects. Sensilla chaetica are located directly on the tip of antennal branches, on the principal antennal axis, and on the back side of the antennal branches. The average number of Sensilla chaetica on each branch is 263 ± 27. They are about 3.5–3.6 μm in width on their basal diameter and around 27–29 μm in length (Table 1). Each sensillum has a thick base with grooves and pointed tip(s). Moreover, there are small spikes (Figure 3D–F) on the tip of each s. chaetica which can be observed at a high-power field of microscopy.

##### Sensilla Coeloconica (SCo)

**Figure 4:**
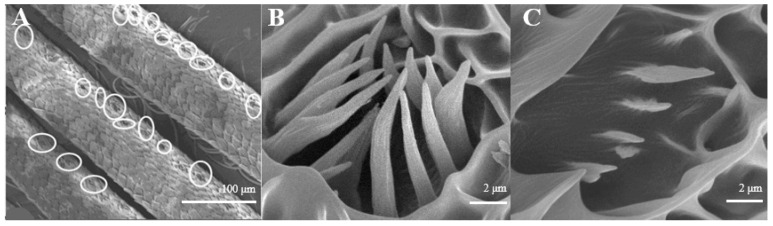
Morphology of sensilla coeloconica in *D. superans*. (**A**) The back side of antennal branches (×400); (**B**) s. coeloconica I (×10,000); (**C**) s. coeloconica II (×10,000).

The average number of Sensilla coeloconica is 163 ± 18 on each branch, which is less than that of the two sensilla types mentioned above. They are mainly distributed on the back side of antennal branches (Figure 4A), which look like chrysanthemum (Figure 4B). There is a hollow and round lumen with an average diameter of 4.5–5.1 μm, created by a cuticle depression and surrounded by some hairs; a wimble stands in the center of the lumen. s. coeloconica are divided into two sub-types, depending on their inner structures of sunken depression:, s. coeloconica I (Figure 4B) are regular, while s. coeloconica II (Figure 4C) are not.

##### Sensilla Gemmiformia (SG)

**Figure 5:**
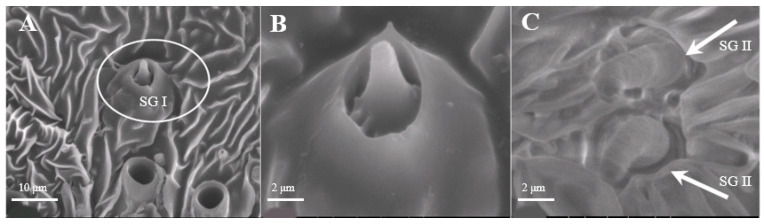
Morphology of sensilla gemmiformia in *D. superans*. (**A**) The principal axis and back side of antennal branches (×2500); (**B**) s. gemmiformia I (×10,000); (**C**) s. gemmiformia II (×10,000).

Sensilla gemmiformia (Figure 5A) are located on the principal axis and back side of antennal branches, and mainly on female antennae. It is tiny in size, has a thick base but a thin flexural tip, and looks like seed sprout in a lumen. On average, the diameter is 2.5–2.7 μm, while the length is 2.8–3.0 μm (Table 1). There are two sub-types according to the shape of the tip: s. gemmiformia I (Figure 5B) and s. gemmiformia II (Figure 5C).

##### Sensilla Basiconica (SB)

**Figure 6:**
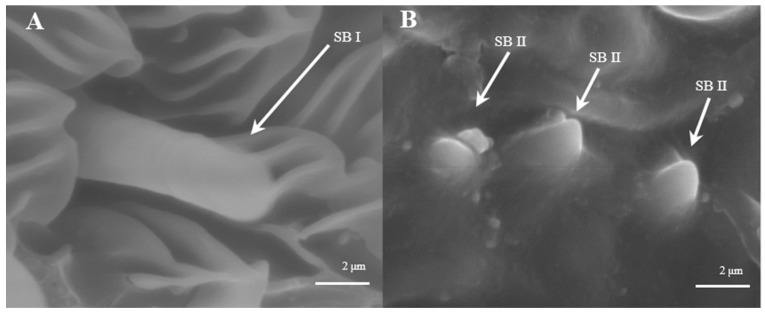
Morphology of sensilla basiconica in *D. superans*. (**A**) s. basiconica I (×10,000); (**B**) s. basiconica II (×10,000).

Sensilla basiconica are located on the reverse side of the principal antennal axis and look like small stingers. They are shorter and more erect than s. trichoidea. The number of s. basiconica is about 2834 ± 157 on the principal antennal axis, which is almost equal to the number of s. trichoideas on four branches. s. basiconica can be divided into two sub-types by their length: s. basiconica I (Figure 6A), which are 3.5–3.6 μm in length, and s. basiconica II (Figure 6B), which are 1.3–1.4 μm in length.

### 3.2. Antennal Morphology and Sensilla Types of L. dispar

**Figure 7:**
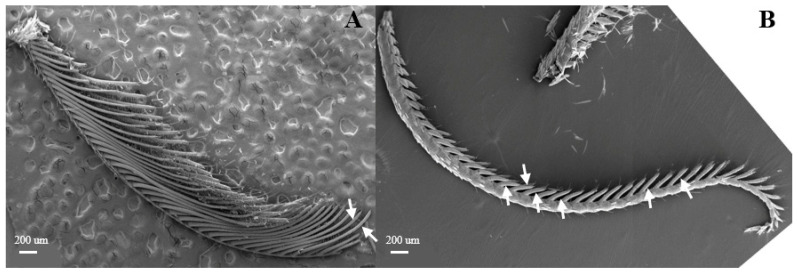
Morphology of *L. dispar* antennae in male (**A**) and female (**B**) moths (×25).

Similar to *D. superans*, the male antennae of *L. dispar* are plumose, and those of females are pectinate. Each flagellum is composed of several sub-segments (Figure 7) in both sexes, and all segments are covered with sensilla, on which pores or squamae were observed.

The male antennal flagellum is shorter than that of females (6.88 and 7.94 μm, respectively), and they consist of 44–47 and 40–44 antennomeres, respectively. The antennal branches are tubular with compartmentation (Figure 7A,B). The antennal branches of males and females are about 0.20 mm and 0.23 mm long, respectively.

In both sexes, the antennal axis has an even diameter throughout its length. There are six different types of antennal sensilla in males: sensilla trichoidea, s. chaetica, s. coeloconica, s. basiconica, s. styloconica, and s. auricillica. And there are seven types of antennal sensilla in females, including the six types found in males plus an additional type, s. squamiform.

#### 3.2.1. Morphology 

##### Sensilla Trichoidea (ST)

**Figure 8:**
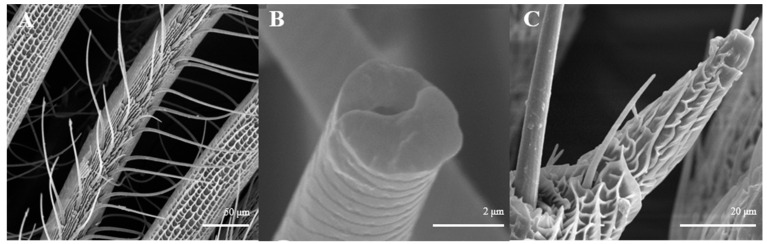
Morphology of sensilla trichoidea in *L. dispar*. (**A**) s. trichoidea on antenna (×500); (**B**) cross-section of s. trichoidea I on antenna (×20,000); (**C**) s. trichoidea II on antenna (×2000).

Sensilla trichoidea are further divided into two sub-types, s. trichoidea I and s. trichoidea II, according to their length and shape. s. trichoidea I (Figure 8A) are 151.5–151.6 μm in length, whereas their basal diameter is 34.1–34.3 μm, standing perpendicularly on the tip of antennal branches in clusters of three sensilla. Cross-striation could be observed clearly on s. trichoidea I under higher magnification (Figure 8B). The s. trichoidea II (Figure 8C) are located on the tip of each female antennal branches or among s. trichoidea I, 31.9–32.1 μm in length and 2.4–2.5 μm of basal diameter. On the female antennae, ten branches with a length of about 300 μm are randomly selected, and the average number of s. trichoidea I and s. trichoidea II on each branch is 87 ± 13 and 31 ± 8, respectively. The overall number of s. trichoidea II is much less than that of s. trichoidea I.

##### Sensilla Chaetica (SC)

**Figure 9:**
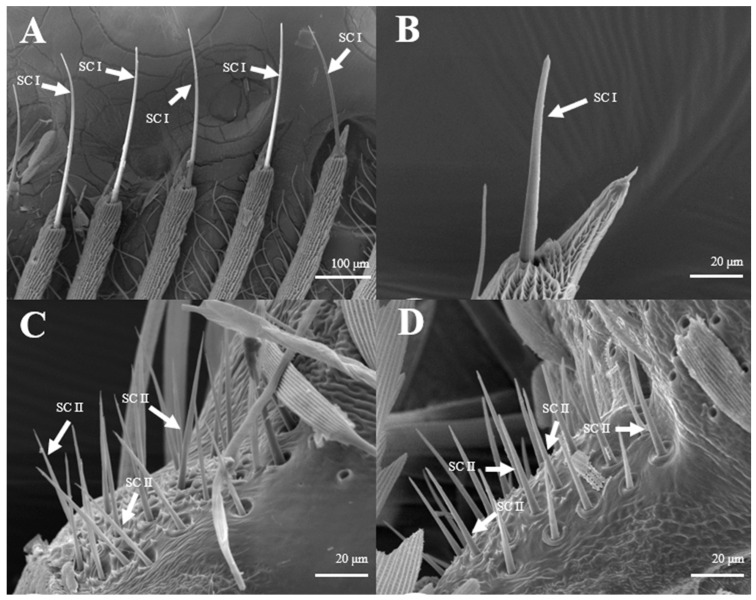
Morphology of sensilla chaetica in *L. dispar*. (**A**) s. chaetica I on the tip of antennal branches of males (×200); (**B**) s. chaetica I on the tip of antennal branches of females (×1000); (**C**) s. chaetica II at base of male antenna (×1000); (**D**) s. chaetica II at base of female antenna (×1000).

Sensilla chaetica on *L. dispar* have two sub-types, s. chaetica I (Figure 9A,B) and II (Figure 9C,D). s. chaetica I are located erectly on the tip of each antennal branch, are 12.0–12.1 μm in basal diameter, and are 281.6–281.7 μm in length in males, while they are 6.53 μm in basal diameter and 76.0–76.2 μm in length in females. s. chaetica II are much shorter than s. chaetica I, and they are broadly localized at base of antennae. On average, the basal diameter is 2.65 μm and the length is 32.8–33.0 μm in both sexes (Table 1).

##### Sensilla Coeloconica (SC)

**Figure 10:**
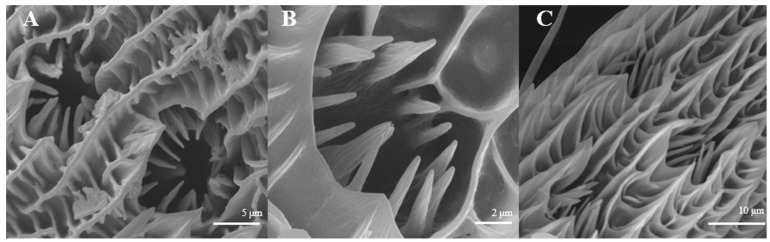
Morphology of sensilla coeloconica in *L. dispar*. (**A**) s. coeloconica of males (×5000); (**B**) s. coeloconica of females (×10,000); (**C**) s. coeloconica of females are clustering (×5000).

There is only one type of antennal sensilla coeloconica in both sexes (Figure 10A,B). They are mainly distributed on the reverse side of antennal branches in a chrysanthemum flower-like pattern. There is a hollow and round lumen, with a diameter of 9.7–9.8 μm on average, created by cuticle depression and surrounded by some hairs; a wimble stands in the center of the lumen. The numbers of s. coeloconica in females are significantly more than those in males (Figure 10C). 

##### Sensilla Styloconica (SSt)

**Figure 11:**
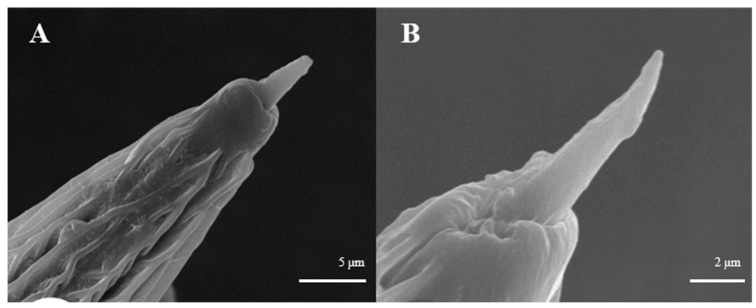
Morphology of sensilla styloconica in *L. dispar*. (**A**) s. styloconica of males (×5000); (**B**) s. styloconica of females (×10,000).

Sensilla styloconica are strong and thumb-like in the basal part. They project from the cuticular wall with a lumen on top of the bulge containing a basiconic prolongation which vary in shape, sometimes spinous, or are thickset and blunt (Figure 11). These sensilla are 6.4–6.5 μm long and 2.3–2.4 μm wide, with wrinkled cuticles. They are often distributed regularly at the distal end of flagellum antennomere in both sexes (Figure 11A,B) (Table 1).

##### Sensilla Basiconica (SB)

**Figure 12:**
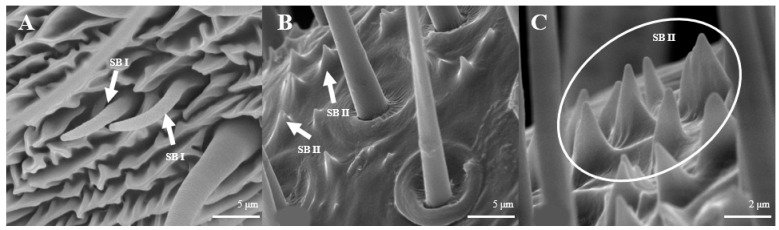
Morphology of sensilla basiconica in *L. dispar*. (**A**) s. basiconica I (×5000); (**B**) s. basiconica II of males (×5000); (**C**) s. basiconica II of females (×10,000).

Sensilla basiconica can be divided into two sub-types by their length. s. basiconica I (Figure 12A), about 9.3–9.4 μm in length, are located on the reverse side of principal antennal axis, while s. basiconic II (Figure 12B,C), about 2.0–2.2 μm long, are distributed mainly at base of flagellum in both sexes and are scattered under s. chaetica II. Three antennas of the female are randomly selected, and the average number of s. basiconica I and s. trichoidea II on each branch is 76 ± 13 and 231 ± 28, respectively. The overall number of s. basiconica I is less than that of s. basiconica II.

##### Sensilla Auricillica (SA)

**Figure 13:**
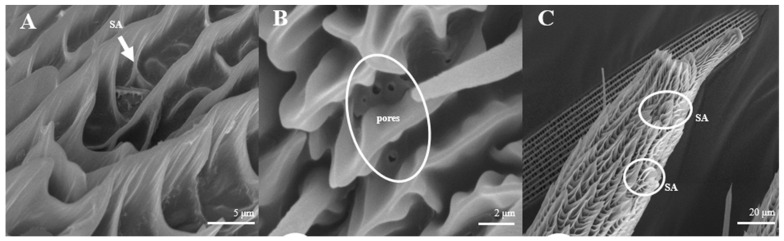
Morphology of sensilla auricillica in *L. dispar*. (**A**) s. auricillica on a male antenna (×5000). (**B**) pores of s. auricillica (×10,000); (**C**) s. auricillica on a female antenna (×1000).

Sensilla auricillica (Figure 13), distributed on the feathery antennal branches in both sexes, are similar to the heart leaf of Gramineae. They are merged with each other on the base and are submerged on the surface. Ear-shaped grooves are seen in the upper part with pores (Figure 13B). The length of male s. auricillica is approximately 4.8–7.5 μm, while the female is 11.4–12.7 μm. The s. auricillica of females (Figure 13C) are longer than those of males (Figure 13A).

##### Sensilla Squamiformia (SSq)

**Figure 14:**
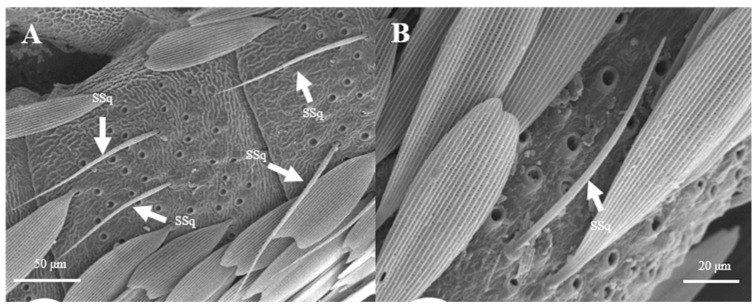
Morphology of sensilla squamiformia in *L. dispar*. (**A**) s. squamiformia (×500); (**B**) s. squamiformia (×1000).

Sensilla squamiformia are elongated or cylindrical, and are scattered among squama (Figure 14). They are about 110.8–111.0 μm in length and 2.5–3.0 μm in width, similar to the dimension of the antennal cuticle. These sensilla were only found on the principal axis of female antennae.

### 3.3. Comparison between the Antennal Sensilla of Two Moths

There are four types of antennal sensilla, trichoidea, chaetica, coeloconica, and basiconica, in common between these two moth species. s. gemmiformia was found in both sexes of *D. superans* but not in *L. dispar*, whereas the opposite is true for s. styloconic, s. auricillica, and s. squamiformia. Among them, s. squamiformia is unique for the females of *L. dispar.*

## 4. Discussion

This is the first detailed report on the morphology of various antennal sensory organ structures of these two economically important forest defoliators, *D. superans* and *L. dispar* adults. The external morphology of antennae, types, and distribution of antennal sensilla in these two moths recorded in this study are similar to those reported for other species of Lepidoptera [28,29,30]. There are both similarities and disparities in antennal sensilla types and distributions among these two species. 

According to SEM, most antennal sensilla of these two moths are located at the principal antennal axis with strong olfactory characteristics (such as s. basiconica and s. styloconica), and on the antennal branches (like s. chaetica and s. coeloconica). s. styloconica, s. auricillica and s. squamiform, not found in the specialist *D. superans,* may be related to the euryphagy of *L. dispar*. There are sexual dimorphisms in the morphology of antennae, sensilla distributions, and sizes in *D. superans*, but not in sensilla types. The number of s. trichoidea is much more in males than that in females, while the opposite is true for s. coeloconica and s. auricillica. Although s. trichoidea differ from s. chaetica due to the lack of the wide socket at their hair bases, these large sharp-tipped hairs also seem to be chemoreceptors [31,32]. These two types of large bristles and hairs have striking differences in their inner structures [33], numbers, and distribution patterns, indicating that they probably have different sensory functions in the moth’s behavior. s. trichoidea II are similar to the s. chaetica I, they all have cuspidal tips and long hairs, but all of the s. chaetica are canulate [34]. In male moths, s. trichoidea reportedly responded to female sex pheromone-related compounds [4,35]. Al-Dosary [36] suggested that s. trichoidea of *Anisolabis maritima* are olfactory sensilla and are involved in the perception of host-related chemical stimuli.

In general, Sensilla chaetica have been named by their various lengths, structures of molar sockets, and characteristics of sharp-tipped hairs [1,37,38,39]; however, the s. chaetica of moths are much more unique with spikes on their tips. We observed that they not only are found on the tip of antennal branches, but also stand on the surface of the main antennal axis, and can be in contact with external odors the earliest; thus, they may operate on recognizing chemical signals from host plants or mates [40,41]. The function of s. chaetica in *Microplitis pallidipes* is considered to be mechanosensory [1], and their function in *Callosobruchus chinensis* and *C. maculatus* is considered to be chemoreceptive [39], similar to that in *L. aeruginosus* and *M. villosus* [42,43]. Uniporous s. chaetica had a combined mechanosensory and gustatory function in *Pallosobruchus chrysocephala* [44]. Different functions among the small pores of *D. superans* are unknown, and more studies need to be carried out.

Sensilla coeloconica have two subtypes, one possessing and the other one lacking costa [45]. It looks the same as s. gemmiformia in the lumen, and is covered with small ciliate, whereas, s. gemmiformia is lubricous. The former kind, which possesses costa, normally occurs in Lepidoptera and Diptera, but is smaller, and is similar to *Helicoverpa armigera* [46], *Culicoidea arakawae*, and *C. schultzei* [47]; it perhaps protects the inner cone from physical damage by the environment. The subtype lacking costa conforms to the other Lepidopteran, such as *Coleophora obducta* [45]. In this study, we found only the subtype possessing costa, with their pattern being a chrysanthemum-flower like on both sexes of these two moths (Figure 4B,C, Figure 10A,B). s. coeloconica are considered to have an olfactory or a contact-chemosensory function, or to be an infra-red sensor [47]. Bruce and Cork [48] even considered that this type of sensilla may function as a receptor of humidity, heat, CO_2_, or plant odor. Whether this has an olfactory or an other sensory function requires further investigation.

S. basiconica, conspicuously short and erect, are also similar to s. trichoidea, but their numbers are fewer than that of s. trichoidea. Some authors assumed that they are most likely olfactory sensilla [43,49,50], but they might function as olfactory or gustatory sensors [51]. Yang et al. [45] showed that s. basiconica of *Coleophora obducta* had a thin cuticle wall surrounding the sensilla lymph lumen with multiple dendrites of sensory neuron cells; these structures typify chemoreceptors. A general odorant-binding protein found in the SB of *Helicoverpa armigera* implies receptivity to host stimulants [52].

Sensilla gemmiformia observed on *D. superans*, but not on *L. dispar*, look similar to s. basiconica, which all have a protrusion in the center, speculating the presence of a sensory centrum. Their function has not yet been completely understood. They were observed sparsely on Coleoptera, such as *Buprestis fairmairei*, and they were supposed to be a mechanoreceptor of gravity [51]. In addition, they are considered to have an acoustic receiving function in Orthoptera [53]. Additionally, in Lepidoptera, this kind of sensilla were found on the antennae of *Polygoniac-aureum*, *Smerinthus planus*, and *Sinopticula sinica*, which also seem to have a mechanosensory function according to their location on the antennal surface [54]. We did not observe the inner structure of s. gemmiformia; further observations are needed in order to understand their function.

s. styloconica are distributed on the tips of antennae of the euryphagous *L. dispar*, but it was not found on the oligophagous *D. superans*. The research by Kvello et al. [55] showed that they can detect and discriminate food sources and/or toxic items. They were also described in many other insects as double-walled and rich in sensory neurons, and involved in both olfaction and gustation [56,57]. Considering the differences of feeding habits of the two kinds of moths, it may suggest that s. styloconica play an important role in insect host locating behavior.

S. auricillica was only found on *L. dispar*. In *Scoliopteryx libatrix*, it could detect host plant volatiles [58]. This kind of sensilla also exists in several euryphagous Lepoidopteran insects such as *Cydia pomonella* and *Spodoptera exigua* [59]. s. auricillica in *Cydia pomonella* have two sub-types, with 3–4 olfactory receptor neurons on each sensillum; single-cell recording showed that they responded to various plant volatiles, and stimulated three excitatory responses of neurons with the same compound. Each sensillum contains a diverse population of ORNs, which often respond to several of the tested compounds [52,60]. So, we infer that s. auricillica in *L. dispar* might also respond to chemical stimuli from different host plants.

S. squamiformia are found frequently among the Lepidopterans, including *Opogona sacchari*, *Hepialus armoricanus*, *Acleris fimbriana*, *Teinopalpus aureus*, *Agathiphaga vitiensis*, *A. queenslandensis*, *Pseudaletia unipuncta*, *Copitarsia consueta*, and *Zamagiria dixolo-phella* [51,61,62,63]. It was also found in *Coleophora obducta* [29]. Yu [64] thought s. squamiformia *Chilo suppressalis* might function in mechanoreception.

## 5. Conclusions

In conclusion, five major types of sensilla were found on both female and male antennae of *D. superans*: s. trichoidea, s. chaetica, s. coeloconica, s. gemmiformia, and s. basiconica, respectively. There are six different types of sensilla on the male antennae of *L. dispar*: sensilla trichoidea, s. chaetica, s. coeloconica, s. basiconica, s. styloconica, and s. auricillica. In addition to the six types found in males, a seventh type of sensilla was only detected on *L. dispar* female antennae, s. squamiformia. Future antennal morphology and electrophysiological studies are needed to confirm the proposed functions of these sensilla types identified in this study. Overall, our data laid a solid foundation for future functional studies in *D. superans* and *L. dispar* using molecular biology, electrophysiology, and anatomy techniques to confirm potential differences in host plant detection between a specialist and a generalist Lepidopteran.

## Figures and Tables

**Table 1:** Sizes and types of antennal sensilla of *D. superans* and *L. dispar*.

Sensilla Types	Sub-Types	*D. superans*		Sub-Types	*L. dispar*	
		Length (μm)	Basal Width (µm)		Length (μm)	Basal Width (µm)
Sensilla trichoidea	s. trichoidea I	62–64	1.3–1.4	s. trichoidea I	151.5–151.6	34.1–34.3
	s. trichoidea II	23–25	2.2–2.3	s. trichoidea II	31.9–32.1	2.4–2.5
Sensilla chaetica	——	27–29	3.5–3.6	s. chaetica I	281.6–281.7 (♂)	12.0–12.1 (♂)
					76.0–76.2 (♀)	6.53 (♀)
				s. chaetica II	32.8–33.0	2.65
Sensilla coeloconica	s. coeloconica I	——	4.5–5.1	——	——	9.7–9.8
	s. coeloconica II					
Sensilla basiconica	s. basiconica I	3.5–3.6	——	s. basiconica I	9.3–9.4	——
	s. basiconica II	1.3–1.4	——	s. basiconica II	2.0–2.2	——
Sensilla gemmiformia	s. gemmiformia I	2.8–3.0	2.5–2.7			
	s. gemmiformia II					
Sensilla styloconica				——	6.4–6.5	2.3–2.4
Sensilla auricillica				——	——	——
Sensilla squamiformia				——	110.8–111.0	2.5–3.0

## Data Availability

We agree to share existing datasets or raw data that have been analyzed in the manuscript, and they will be made available to other researchers following publication. Data availability status: All data are contained within the article.
